# Nurses' knowledge and attitudes toward pediatric pain assessment and management: a scoping systematic review from 2015 to 2025

**DOI:** 10.3389/frhs.2026.1918667

**Published:** 2026-08-27

**Authors:** Haytham Al-Oran, Yousef Rubbai

**Affiliations:** 1 Department of Child Health Nursing, Faculty of Nursing, Mutah University, Al-Karak, Jordan; 2 Department of Adult Health Nursing, Faculty of Nursing, Al-Hussein Bin Talal University, Ma'an, Jordan

**Keywords:** attitude, children, knowledge, nursing, pain assessment, pain management

## Abstract

**Introduction:**

Nurses play an essential role in providing care to patients, and having the necessary skills, knowledge, and attitudes for effective pain assessment and treatment is crucial for ensuring best patient outcomes. The purpose of this paper is to review recent research about current nurses' knowledge and attitude toward pediatric pain assessment and management in pediatric settings.

**Method:**

The methodological approach taken in this paper is a scoping review using Arksey and O'Malley's five-step framework (2005). Databases including EBSCO host, PubMed, CINAHL, Springer, Cochrane, Science Direct, Ovid, and Google Scholar were searched to collect full-text articles available in English between 2015 and 2026. 20 out of 235 full-text papers met the inclusion criteria in the current scoping review.

**Results:**

The synthesis of the results of the 20 included research studies revealed that nurses worldwide had poor knowledge and attitudes concerning pediatric pain assessment and management. Various tools were utilized to assess nurses' knowledge and attitudes. The majority of research adopted the use of true/false or multiple-choice questions, as well as Likert scales with acceptable validity and reliability. The most extensively utilized tool was the Knowledge and Attitudes Survey Regarding Pain.

**Conclusion:**

This review's findings imply that pain education programs should be refined to enhance nurses' knowledge and attitudes toward pediatric pain assessment and management. Studies in the future should focus on recognizing individual and environmental features that influence nurses' level of knowledge and attitudes to improve the development of educational programs and the quality of care provided.

## 1 Introduction

The International Association for the Study of Pain (IASP) defines pain as “an unpleasant sensory and emotional experience associated with actual or potential tissue damage” (1). Pain is one of the most common symptoms related to the health conditions that nurses encounter every day. Pain is a significant stressor that affects a person's physiological, psychological, and emotional wellbeing, despite its role as a protective mechanism prompting individuals to seek healthcare services (2). Untreated pain can cause physiological and behavioral stress reactions that influence every system in the patient's body, resulting in adverse outcomes (3).

Pain is a worldwide health problem and a universal human experience that affects individuals of all races, genders, age groups, geographical areas, and socioeconomic statuses (4, 5). In recent years, pain has become an important topic of research, making pain assessment and management a key goal in healthcare (6–10). Despite substantial advances in knowledge, the accessibility of guidelines, and developments in technologies and pharmacologic and non-pharmacologic pain treatment approaches, pain continues to be underestimated and undertreated, with hospitalized patients frequently experiencing excessive pain (8, 9).

Children range in age from preterm infants to adolescents. Age-related changes in children are evident, affecting pain assessment and treatment due to variations in physiology and pharmacology compared with adults (11). Pain in children can be measured based on age, communication ability, self-report, observation of behavior, or physiological indicators. Fenta et al. (11) pointed out that nurses are usually responsible for assessing and treating children's pain. Incorrectly diagnosed and inadequately treated pain can have a severe influence on a child's development, increase psychological discomfort (e.g., fear and anxiety), and potentially lead to chronic pain that persists into adulthood (12, 13). As a result, nurses should have a solid foundation of knowledge about pediatric pain assessment and management, along with an appropriate attitudes toward therapy that reduces suffering and improves overall patient outcomes.

Nurses, as the primary care providers at the bedside, play a crucial role in assessing and administering pain relief treatments, making their expertise essential for efficient pediatric pain management (9). As experts in pain assessment, management, and patient education, nurses play an important role in alleviating the patient's pain across healthcare settings (7, 14). To accurately assess and effectively manage pain, nurses must gain current knowledge, have appropriate attitudes, and possess competent skills (15).

Nurses should use a methodical approach with a reliable and valid tool for accurate pain assessment, verify and confirm the pain level reported by the patients themselves, provide support, utilize pharmacological and non-pharmacological approaches to pain management, and assess management effectiveness (6, 16). Nurses manage acute and chronic pain and evaluate the effectiveness of medical treatments. However, previous research has revealed gaps in knowledge, attitude, and practices regarding pediatric pain assessment and management (6, 11, 17–19). Consequently, pain is often misinterpreted, underestimated, and mismanaged in many different healthcare settings throughout the world.

Therefore, addressing gaps in nurses' knowledge and attitudes about pediatric pain is crucial to prevent inadequate pain control, which can cause unnecessary suffering, prolonged hospital stays, and increased healthcare expenditures. To date, no scoping review has been published on nurses' knowledge and attitudes toward pediatric pain assessment and management in pediatric settings. This review, therefore, a clear need for a review that examines and maps the available evidence on nurses' knowledge and attitudes toward pediatric pain assessment and management in pediatric settings. This review contributes to the literature by systematically analyzing studies on nurses' knowledge and attitudes about pediatric pain assessment and management, synthesizing existing findings and identifying significant gaps in both knowledge and practice. Thus, the present review aimed to synthesize and critically appraise research conducted between 2015 and 2025 on nurses' knowledge and attitudes toward pediatric pain assessment and management in pediatric settings.

## 2 Methods

The scoping review method is useful when addressing extensively studied topics, as it enables mapping of the literature, identification of major concepts, and recognition of areas for further study. The authors followed the scoping review approach as outlined by Arksey and O'Malley (20). Arksey and O'Malley (20) classified the process into five phases, which are described in the following.

### 2.1 Step 1: Identify the review question

Arksey and O'Malley (20) recommend beginning with a general question and then narrowing the focus to capture the scope and breadth of the literature. This review attempted to answer the following review question: What is the level of nurses' knowledge and attitudes toward pediatric pain assessment and management in pediatric settings?

### 2.2 Step 2: Identifying relevant studies

A systematic search was conducted to capture the indexed papers related to the topic using major electronic medical databases. These databases included EBSCOhost, PubMed, Cumulative Index of Nursing and Allied Health Literature (CINAHL), Springer, Cochrane, Science Direct, Ovid, and Google Scholar. These databases were selected for their relevance and coverage of relevant papers in key fields. The search strategy was designed to find studies that addressed key concepts in the assessment and management of pediatric pain, focusing particularly on nurses. The search comprised four steps: (1) define the problem associated with the research questions, (2) conduct a literature search, (3) review and screen eligible articles, and (4) organize and critically analyze the articles.

The search used a combination of free-text keywords, medical subject headings (MeSH), and Booleans (OR, AND) to retrieve all relevant papers. Search keywords included “attitude,” “children,” “knowledge,” “nursing,” “pain assessment,” and “pain management.” In addition, Boolean operators were employed to combine the search key terms: “nurse” OR “pediatric nurse,” “nursing knowledge” OR “pediatric nurse knowledge,” AND “nursing attitude” OR “pediatric nurse attitude,” AND “pain assessment,” AND “pain management” OR “pain treatment,” AND “children” OR “pediatric.”

### 2.3 Step 3: Study selection

Inclusion and exclusion criteria are essential to filter the number of papers generated and confirm relevancy. As recommended by Arksey and O'Malley (20), the inclusion and exclusion criteria were established after thoroughly familiarizing with the available literature. The inclusion criteria were as follows: papers published between 2015 and 2025 (the search period was chosen due to provide a sufficient time frame to collect enough scholarly articles and reflect the current state of the literature), full-text papers available in English, papers addressing nurses' knowledge and attitudes as the primary outcomes regarding pediatric pain assessment and management, and both quantitative and qualitative studies. Exclusion criteria were as follows: gray literature, abstract only publications, letters and case reports, editorial or opinion-based articles, and papers that focused on healthcare professionals other than nurses. The inclusion and exclusion criteria for all papers were agreed upon based on consensus between the authors.

The screening for eligibility process started with a preliminary screening of titles and abstracts (stage 1), followed by removal of duplicates across different electronic databases (stage 2). The authors then separately checked stage 1 and stage 2 papers, after which the eligible full-text publications (stage 3) were retrieved and once again thoroughly checked against the inclusion and exclusion criteria. All the papers were examined by the first author for criteria, and the decisions made were then reviewed with the second author. Next, both authors thoroughly reviewed and examined the relevant papers (stage 4), with detailed notes taken to synthesize the key ideas, including study aims, methods, instruments, and main results.

**Figure 1:**
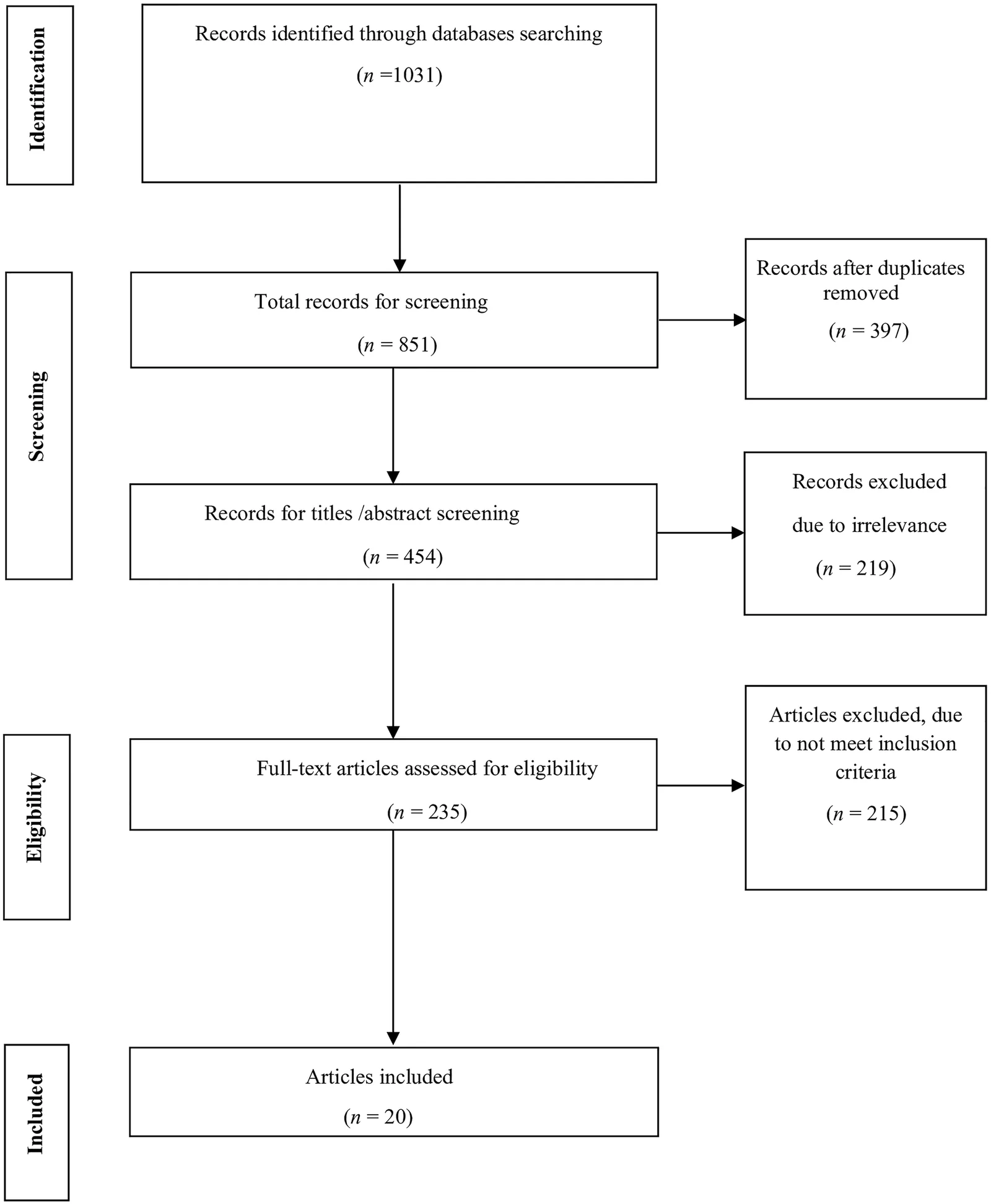
PRISMA flowchart.

The search strategy yielded 1,031 relevant papers (187 from EBSCO host, 71 from PubMed, 51 from CINAHL, 62 from Springer, 19 from Cochrane, 63 from Science Direct, 33 from Ovid, and 545 from Google Scholar). Of these, 397 articles were found to be duplicates, and 219 were excluded due to irrelevance to the present scoping review. A total of 235 full-text papers underwent eligibility assessment. Of these, we excluded 215 due to not meeting inclusion criteria. In the end, 20 full-text studies were identified as relevant to the current scoping review (Figure 1).

### 2.4 Step 4: Charting the data

The first and second authors independently examined the quality of all included studies employing the Joanna Briggs Institute (JBI) critical appraisal approach. Methodological quality and analytical rigor were assessed for the 20 papers identified during the search process. The included studies were evaluated using a cross-sectional survey appraisal checklist comprising eight items. Every assessment criterion included four answers to each question: “Yes,” “No,” “Unclear,” and “Not applicable.” According to the JBI Critical Appraisal Tool, the quality of the included studies ranged from 75% to 100%. The scoping review guidelines template was employed to extract data from the included studies. A data extraction table matrix was designed to collect data from the each study. Table 1 includes information such as the author's name, publication year, study country, study design, study population, study tool used, and results.

**Table 1:** Summary of the articles included in the review.

Author(s)	Country	Study design	Study population (*n*)	Study tools	Results
Ortiz et al. (31)	Mexico	Cross-sectional	Nurses (111)	PNKAS	Among nurses, knowledge and attitudes about pediatric pain assessment and management were poor (mean score = 40.1%), with scores ranging from 22.5 to 62.5
Bajjali (26)	Palestine	Cross-sectional	Nurses (256)	PNKAS	Nurses had insufficient knowledge and attitudes toward pain assessment and management, with a mean score of 50.4% and correct answers ranging from 84.8% to 25%
Smeland et al. (30)	Norway	Cross-sectional	Nurses (193)	PNKAS—Norwegian Version	Nurses had poor knowledge and attitudes in relation to pediatric pain assessment and management, with a mean score of 28.8% and correct answers ranging from 35% to 100%
Peirce et al. (29)	Australia	Cross-sectional	Nurses (590)	Pediatric Pain Knowledge and Attitudes Questionnaire (Revised)	Nurses had higher levels of knowledge and attitudes toward pediatric pain assessment and management. Knowledge and attitude mean scores were 77.6% and 72.46%, respectively
Notejane (27)	Uruguay	Cross-sectional	Nurses (151)	PNKAS	Nurses' mean score of knowledge and attitude of pain assessment and management was 61%. The study revealed that nurses had acceptable levels of knowledge and attitudes regarding pediatric pain assessment and management as compared with other studies
Alotaibi (25)	Saudi Arabia	Cross-sectional	Nurses (410)	PNKAS—Shriners Revision	With mean scores of 45.2%, pediatric nurses showed poor overall knowledge and attitudes regarding pediatric pain assessment and management
Kusi Amponsah et al. (28)	Ghana	Cross-sectional	Nurses (65)	PNKAS	Nurses had insufficient knowledge and attitudes regarding pediatric pain assessment and management, with a mean correct score of 6.9% and correct answers ranging from 21.4% to 57.1%
Hua et al. (23)	China	Cross-sectional	Nurses (2,882)	PNKAS	Nurses did not have adequate levels of knowledge and attitudes toward pediatric pain management. The total mean score on PNKAS was 14.88 ± 3.58
Dana and Tefera (21)	Ethiopia	Cross-sectional	Nurses (94)	PNKAS—Shriners Revision	Nurses had inadequate knowledge and attitudes toward pediatric pain assessment and management. Nurses' mean knowledge and attitude score of pediatric assessment and pain management was 57.27%
Tadesse et al. (5)	Ethiopia	Cross-sectional	Nurses (226)	KASRP	Nurses had poor levels of knowledge toward pediatric pain assessment and management, with a means score of = 42.5%
Li et al. (24)	China	Cross-sectional	Nurses (293)	PNKAS	Nurses had insufficient levels of knowledge and attitudes regarding pediatric pain management
Lulie et al. (16)	Ethiopia	Cross-sectional	Nurses (267)	PNKAS	Nurses had adequate levels of knowledge and attitudes regarding pediatric pain management
Tsuboi et al. (13)	Japan	Cross-sectional	Nurses (265)	PNKAS	With a mean score of 31.9%, the study indicated that pediatric nurses have insufficient knowledge and attitudes toward pediatric pain assessment and management
Fenta et al. (11)	Ethiopia	Cross-sectional	Nurses (292)	PNKAS	Nurses had poor knowledge and attitudes regarding pediatric pain assessment and management. The total mean score was 43.1%, ranging from 28.6% to 61.9%
Tagele et al. (22)	Ethiopia	Cross-sectional	Nurses (234)	PNKAS	Nurses had adequate levels of knowledge and attitudes regarding pediatric pain assessment and management
Mekonen et al. (10)	Ethiopia	Cross-sectional	Nurses (266)	PNKAS	Knowledge and attitudes toward pediatric pain assessment and management were insufficient among more than half of the participants
Shrestha-Ranjit et al. (18)	Nepal	Cross-sectional	Nurses (116)	PNKAS	Nurses reported significant deficits of knowledge and attitudes toward pediatric pain assessment management, with a mean score of 37.53%, ranging from 14% to 56%
Shanmugam et al. (19)	Saudi Arabia	Cross-sectional	Nurses (119)	PNKAS	Mean score of correct answers for all nurses was 36.59%, ranging from % 2.5 to 86.5%. The participants had consistently poor levels of knowledge and attitudes toward pediatric pain assessment and management
Farhad and Iftikha (9)	Pakistan	Cross-sectional	Nurses (165)	KASRP	Nurses had low levels of knowledge and attitudes regarding pediatric pain assessment and management
Alrusan et al. (17)	Jordan	Cross-sectional	Nurses (61)	KASRP	Nurses had insufficient knowledge and attitudes toward pediatric pain assessment and management, with a mean score of 56.2%

PNKAS, pediatric nurses’ knowledge and attitude survey regarding pain; KASRP, knowledge and attitudes survey regarding pain.

### 2.5 Step 5: Collate, summarize, and report the data

In this scoping review, all full-text papers that were published in the English language between 2015 and 2025 were included (Table 1).

## 3 Results

As shown in Table 1, the studies included span from 2015 to 2025 and originate from various countries, including Mexico, Palestine, Norway, Australia, Uruguay, Saudi Arabia, Ghana, China, Ethiopia, Japan, Nepal, Pakistan, and Jordan. All the reviewed research employed cross-sectional designs. There were 20 cross-sectional designs, and none of these studies applied mixed-methods or qualitative approaches. With regard to the study setting, six were conducted in Ethiopia (5, 10, 11, 16, 21, 22) and two were carried out in China (23, 24) and Saudi Arabia (19, 25). One study each was performed in Jordan, Palestine, Pakistan, Ghana, Uruguay, Mexico, Australia, Japan, Norway, and Nepal (9, 13, 17, 26–31).

As for the year of publication, one each was published in 2015 (31) and 2021 (21). Three studies were published in 2018 (26, 29, 30), 2022 (5, 16, 24), and 2023 (11, 13, 22). Four studies were published in 2019 (23, 25, 27–29), and five studies were published in 2025 (9, 10, 17–19). With respect to the sample size, all descriptive studies included relatively small-to-large sample sizes: Hua et al. (23) (*n* = 2,882), Peirce et al. (29) (*n* = 590), Alotaibi et al. (25) (*n* = 410), Li et al. (24) (*n* = 293), Fenta et al. (11) (*n* = 292), Mekonen et al. (10) (*n* = 266), Lulie et al. (16) (*n* = 267), Tsuboi et al. (13) (*n* = 265), Bajjali (26) (*n* = 256), Tagele et al. (22) (*n* = 234), Tadesse et al. (5) (*n* = 226), Smeland et al. (30) (*n* = 193), Farhad and Iftikha (9) (*n* = 165), Notejane et al. (27) (*n* = 151), Shanmugam et al. (19) (*n* = 119), Shrestha-Ranjit et al. (18) (*n* = 116), Ortiz et al. (31) (*n* = 111), Dana and Tefera (21) (*n* = 94), Kusi Amponsah et al. (28) (*n* = 65), and Alrusan et al. (17) (*n* = 61). In terms of study participants, all studies included pediatric nurses who working in pediatric settings.

As for study instruments, the reviewed studies did not employ the same questionnaire to assess nurses' knowledge and attitudes. Three main tools were used in the reviewed studies. The KASRP tool was utilized to assess the level of nurses' knowledge and attitudes toward pediatric pain assessment and management by three studies (5, 9, 17). One study used the revised version of the Pediatric Pain Knowledge and Attitudes Questionnaire (29). However, the majority of the reviewed studies utilized the pediatric nurses’ knowledge and attitude survey regarding pain (PNKAS) tool. All these tools are psychometrically verified; nevertheless, employing multiple questionnaires in review studies presents challenges for precise evaluation of nurses' knowledge and attitudes regarding pediatric pain assessment and management.

## 4 Discussion

To the authors' knowledge, this review is the first attempt to assess up-to-date evidence regarding nurses' knowledge and attitudes toward pediatric pain assessment and management. Effective pediatric pain management involves sufficient information, appropriate attitudes, and competent assessment skills. Nurses are responsible for assessing pain and employing both pharmacologic and non-pharmacologic interventions for pediatric patients. In the present review, findings from the majority of included studies revealed that nurses demonstrated poor levels of knowledge and attitudes toward pediatric pain assessment and management. This evidence suggests that nurses lack sufficient understanding of how to help pediatric patients maintain their sense of comfort or even how to effectively manage their discomfort.

Pediatric pain assessment and management must be grounded in a solid scientific basis, supported by appropriate attitudes, and offered with a commitment to patient-centered treatment. Several attempts have been made to examine nurses' knowledge and attitudes toward pediatric pain assessment and management. In the context of pediatric services, when delivering care to children and their parents, nurses should integrate key elements of family-centered care, ensuring that healthcare is planned not only for the child but also for all members of the family who serve as care recipients (32).

Peirce et al. (29) found that nurses achieved high levels of knowledge and attitudes toward pediatric pain assessment and management, with overall mean scores of 77.6% and 72.46%, respectively, indicating that they regularly apply pain assessment and management to pediatric patients in their clinical setting. Other studies reported similar findings. For example, a cross-sectional study conducted by Notejane et al. (27) revealed that nurses had acceptable levels of knowledge and attitudes about pediatric pain assessment and management as compared with other studies, with overall mean scores of 61%. Descriptive data confirmed these findings and highlighted areas where nurses demonstrated adequate levels of knowledge and attitudes toward pediatric pain assessment and management (16). These findings are in the same vein as those reported in a more recent study (22). Such results suggest that nurses are prepared to deliver nursing care with greater efficacy. An important factor that prepares nurses to provide the best-quality pain assessment and management is the curriculum in nursing schools. It is crucial to note that attitudes developed from a comprehensive knowledge of pain and its treatment form the cornerstones of effective pain management.

Conversely, as Ortiz and colleagues (31) found upon assessing the levels of knowledge and attitudes about pediatric pain assessment and management, nurses who working in pediatric settings reported low levels of knowledge and attitudes, with a mean score of 40.1%. To some extent, these results are consistent with the findings obtained by Smeland et al. (30) and Bajjali (26), indicating that nurses had insufficient knowledge and attitudes toward pediatric pain assessment and its management. Ultimately, these knowledge gaps and negative attitudes may affect how well children who are in pain are treated in pediatric settings. Poor understanding of pediatric pain assessment, as previously mentioned, results in undertreatment of children's pain, impacting the patient's quality of care.

These results parallel the findings of other studies (26, 28, 32). For example, Dana and Tefera (21), Li et al. (24), Tadesse et al. (5), and Tsuboi et al. (13) reported that pediatric nurses have insufficient knowledge and attitudes toward pediatric pain assessment and management. In line with these observations, Fenta et al. (11) emphasized that nurses in such settings did not consistently follow protocols for pediatric pain assessment and management. The results of this study (11) are consistent with those of earlier research, highlighting persistent concerns about nurses' insufficient knowledge and attitudes toward children in pain. Limited availability of pain assessment tools, inadequate treatment equipment, lack of designated areas for documenting pain, and insufficient institutional policies or guidelines for pediatric pain assessment and management all represent obstacles to achieving adequate knowledge and attitudes among nurses who are working in pediatric settings.

In line with previously reviewed studies, Mekonen et al. (10) employed a cross-sectional design and found that knowledge and attitudes toward pediatric pain assessment and management were insufficient among more than half of the participants. This result was once again consistent with findings from other descriptive studies (9, 17, 23, 29). These studies revealed a lack of knowledge and attitudes toward pain in several areas linked to pain assessment and management. The main areas that demonstrated the greatest knowledge gaps and inadequate attitudes were (1) medication-based knowledge, (2) insufficient pain assessment and treatment skills, and (3) inadequate awareness of non-pharmacological therapies. Nurses should receive more comprehensive education about the use of opioid analgesic medications and the major side effects of opioid drug therapy in children. Besides that, nurses should place greater emphasis on self-reported pain assessment and take it into consideration.

Taken together, the present review revealed that nurses working in pediatric settings reported significant deficits in knowledge and attitudes toward pediatric pain assessment and management. The literature suggests that nurses infrequently implemented pain assessment and management protocols for pediatric patients in clinical settings. This review adds considerably to future research by providing a foundation for the development of targeted educational programs aimed at improving nurses' knowledge and attitudes toward pediatric pain assessment and management.

### 4.1 Implications for nursing education, clinical practice, and future research

This review highlights a variety of key results that carry implications for nursing education, health institutions, and research. These findings emphasize the necessity of providing ongoing training courses in pain assessment and management that integrate theoretical and clinical methods. Continuing training for nurses is essential to maintain up-to-date current knowledge and skills about pain assessment and management. Periodic education that accounts for varying levels of professional experience and different healthcare contexts can foster more consistent evidence-based practices across healthcare organizations, thereby ensuring the highest-quality pain assessment and treatment for pediatric patients. Healthcare policymakers should also develop and implement policies and guidelines across organizations to guide nursing practices and guarantee a consistently high level of patient care. As for research implications, this review may contribute to filling gaps in knowledge and enriching the literature, while also providing insightful information to guide future research. It is also important to assess the impact and long-term efficacy of educational interventions on nurses' knowledge and attitudes relating to pediatric pain assessment and management.

### 4.2 Limitations

There are some limitations to this review. First, only papers published in English were included in the current review. There might be papers published in different languages that may have offered insightful perspectives on the topic of study. Second, the present review focused exclusively on nurses, constraining the extent to which the results reflect the level of knowledge and attitudes toward pain assessment and management of other health professions. Finally, the homogeneity of study designs hindered deeper analytical synthesis and restricted cross-study comparability.

### 4.3 Recommendations for nursing education, clinical practice, and future work

Nursing faculties should review the content of nursing curricula to ensure that it reflects contemporary standards of practice in terms of depth, accuracy, and relevancy of pain assessment and treatment. According to the present review, comprehensive and thorough educational programs must be designed to address the particular needs of nurses at all nursing levels. Both undergraduate and graduate nursing core curricula should be thoroughly reviewed to ensure that the educational modules provide appropriate, relevant, and applicable knowledge, thereby preparing nurses to assess and manage pain among pediatric patients. In this context, adopting a holistic approach to nursing education that targets pain assessment and management provides nurses with the opportunities to engage in specialized courses and apply reflective learning interventions.

Create and integrate directly several strategies is needed to improve nurses' comprehension and practice through standard education programs and ongoing clinical training. More specialized endeavors, including quality improvement programs, should be implemented at healthcare institutions. Such endeavors might involve a variety of strategies to advance the understanding and enhance the methods of pain assessment and management. Besides this, advancing equitable pediatric health in alignment with broader worldwide health goals will need ongoing international collaboration and the continued implementation of nursing research. The insights of the present review highlight challenges in implementation requiring further investigation using mixed-methods or qualitative research to capture more specific themes and deeper insights. Furthermore, longitudinal research is recommended to evaluate how nurses' knowledge and attitudes about pain assessment and management evolve over time in response to focused training courses or exposure to standard clinical practices.

## 5 Conclusions

Pediatric pain assessment and management have gained significant attention in both Western and non-Western nations, with evidence linking practices to improved outcomes for hospitalized children. In this review, we identified that the knowledge levels and attitudes of nurses remain inadequate and insufficient. The findings indicate that nurses have significant knowledge gaps and inaccurate assumptions that might affect how well they manage pediatric patients and how comfortable the patients are. The present paper highlights the urgent need for integrated, contextualized methods that boost intuitional commitment and clinical capacity to improve pediatric pain care. A multidimensional approach is needed to fill these gaps, including reform of the curriculum, continuous education, and improvements of institutional policies. By improving nurses' knowledge and attitudes regarding pediatric pain assessment and management, healthcare systems can better serve the needs of pediatric patient and enhance quality-of-care outcomes.

## Data Availability

The dataset presented in the study is available on request from the corresponding author during submission or after publication.
